# Supplementary material for: A proteomic platform to identify off-target proteins associated with therapeutic modalities that induce protein degradation or gene silencing
Source: Sci Rep. 2021 Aug 4;11:15856. doi: 10.1038/s41598-021-95354-3 (PMC8338952; doi:10.1038/s41598-021-95354-3)

**Supplementary Information (SI)**

A Proteomic Platform to Identify Off-Target Proteins Associated with Therapeutic Modalities that Induce Protein Degradation or Gene Silencing

Xin Liu^†,1^, Ye Zhang^†,2^, Lucas D. Ward^†,3^, Qinghong Yan^†,4^, Tanggis Bohnuud^†,5^, Rocio Hernandez^†,6^, Socheata Lao^†,7^, Jing Yuan^†, 8^, and Fan Fan^†.7,*^

^†^Amgen Inc., Translational Safety & Bioanalytical Sciences, 360 Binney St., Cambridge, MA02142

^1^Current address: Novartis Institutes for Biomedical Research, 500 technology square, Cambridge, MA 02139

^2^Current address: Amgen Inc., Genome Analysis Unit, 1120 Veteran Blvd., South San Francisco, CA94080

^3^Current address: Alnylam Pharmaceuticals, 300 Third St., Cambridge, MA 02142

^4^Current address: Fosun Pharma, 104 Carnegie Center Drive, Suite 204, Princeton, NJ 08540

^5^Current address: Beam Pharmaceuticals, 26 Landsdowne St., Cambridge, MA 02139

^6^Current address: Amgen Inc., Translational Safety & Bioanalytical Sciences, 1 Amgen Center Dr., Thousand Oaks, CA91320

^7^Current address: Amgen Inc., Translational Safety & Bioanalytical Sciences, 1120 Veteran Blvd, South San Francisco, CA94080

^8^Current address: Drug Safety Research and Development, Pfizer Inc., 1 Portland St., Cambridge, MA 02139

^*^Corresponding Author: email: ffan@amgen.com; Tel: +1(617)444-5187

**Supplementary Figure 1**

Venn diagram safety proteome composition in CV, CNS, and respiratory (gene centric).


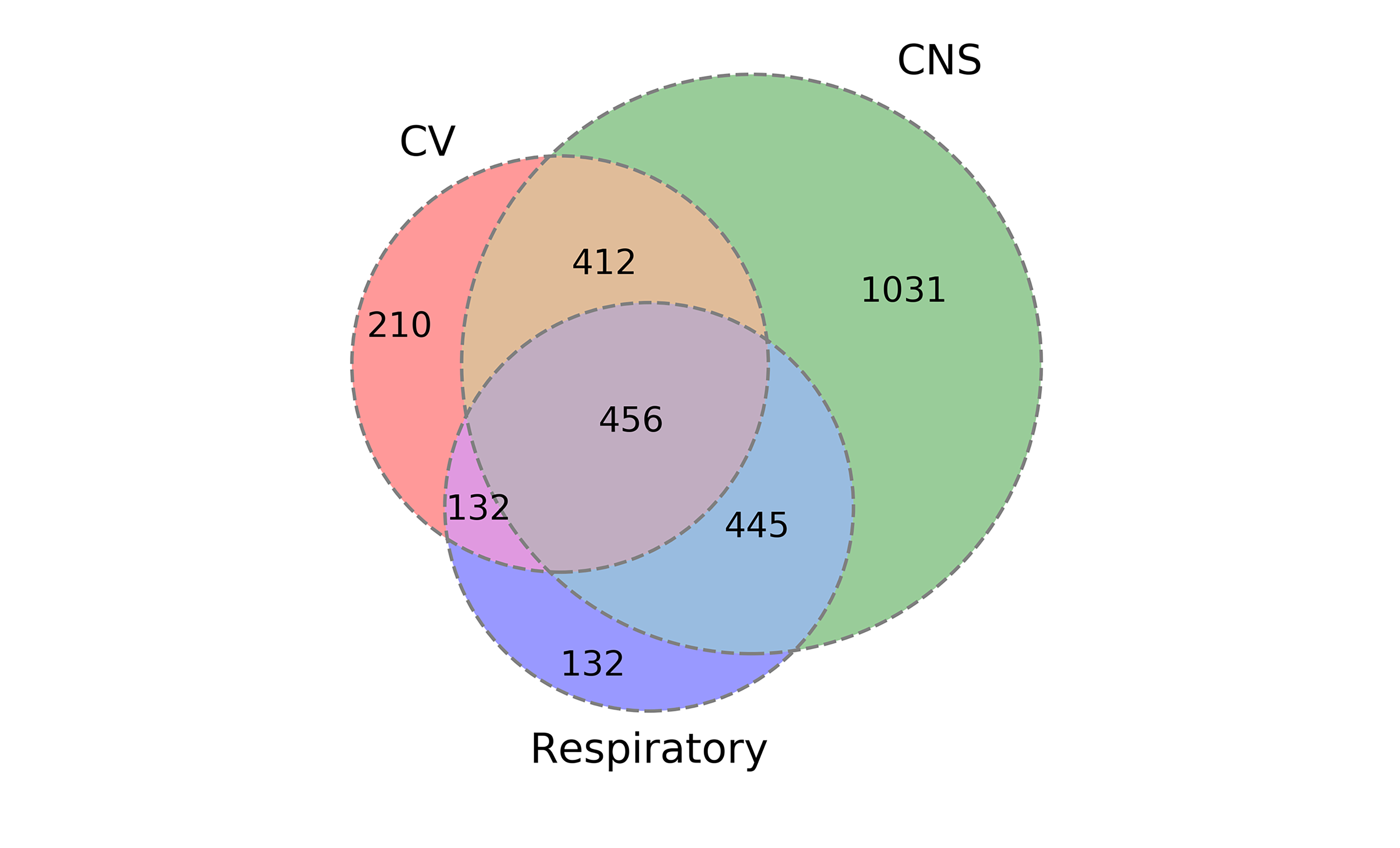


**Supplementary Figure 2**

Coverage of the human genome by chromosomes. Distributions of the quantified proteins (gene centric) are shown in red (cellular proteins) and green (secreted proteins), safety proteome are shown in orange versus total genes (blue) for each chromosome.


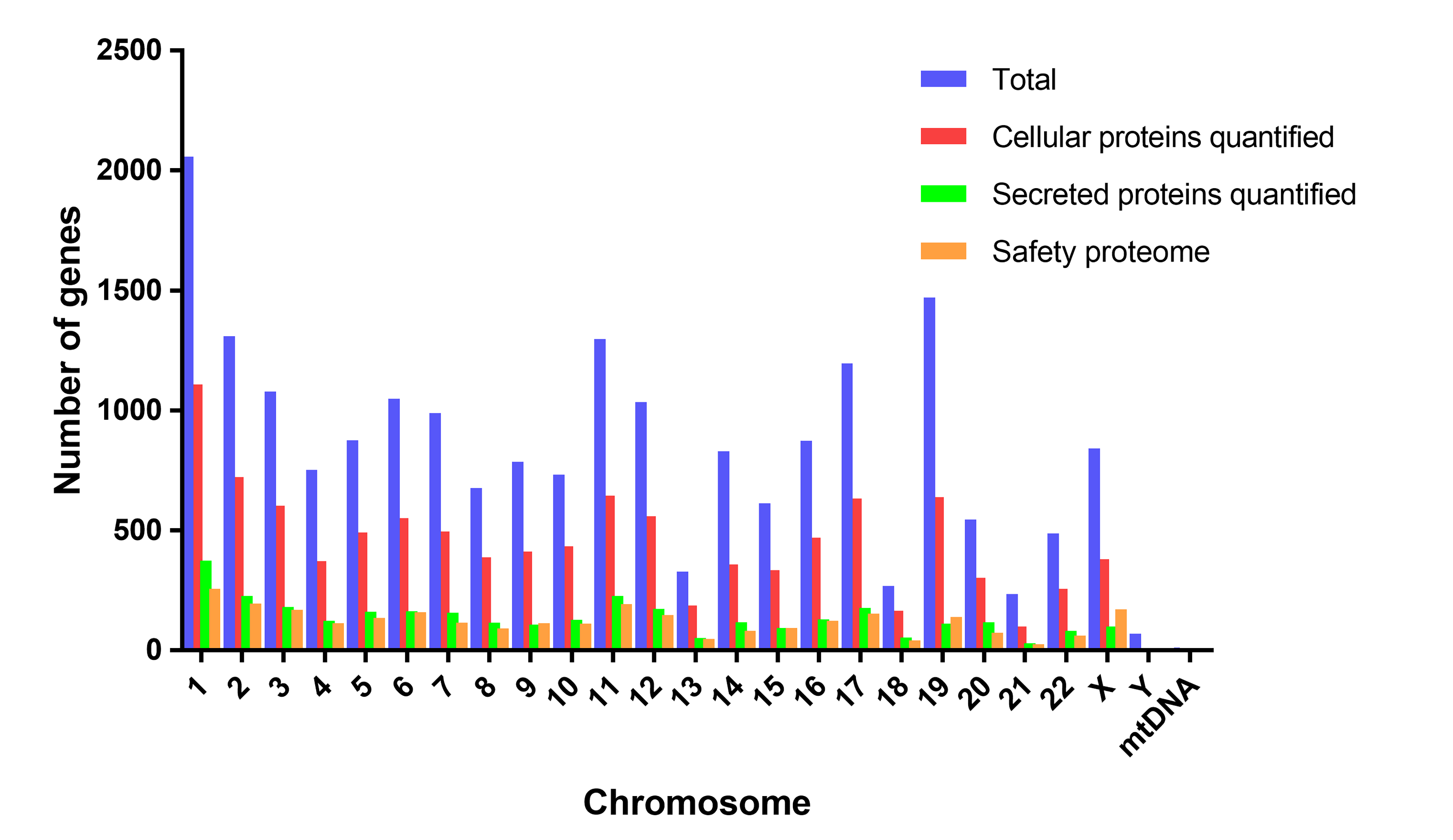


**Supplementary Figure 3**

Composition of the secretomes (quantified proteins). Venn diagram (A) and pie chart (B) of secretion pathways.


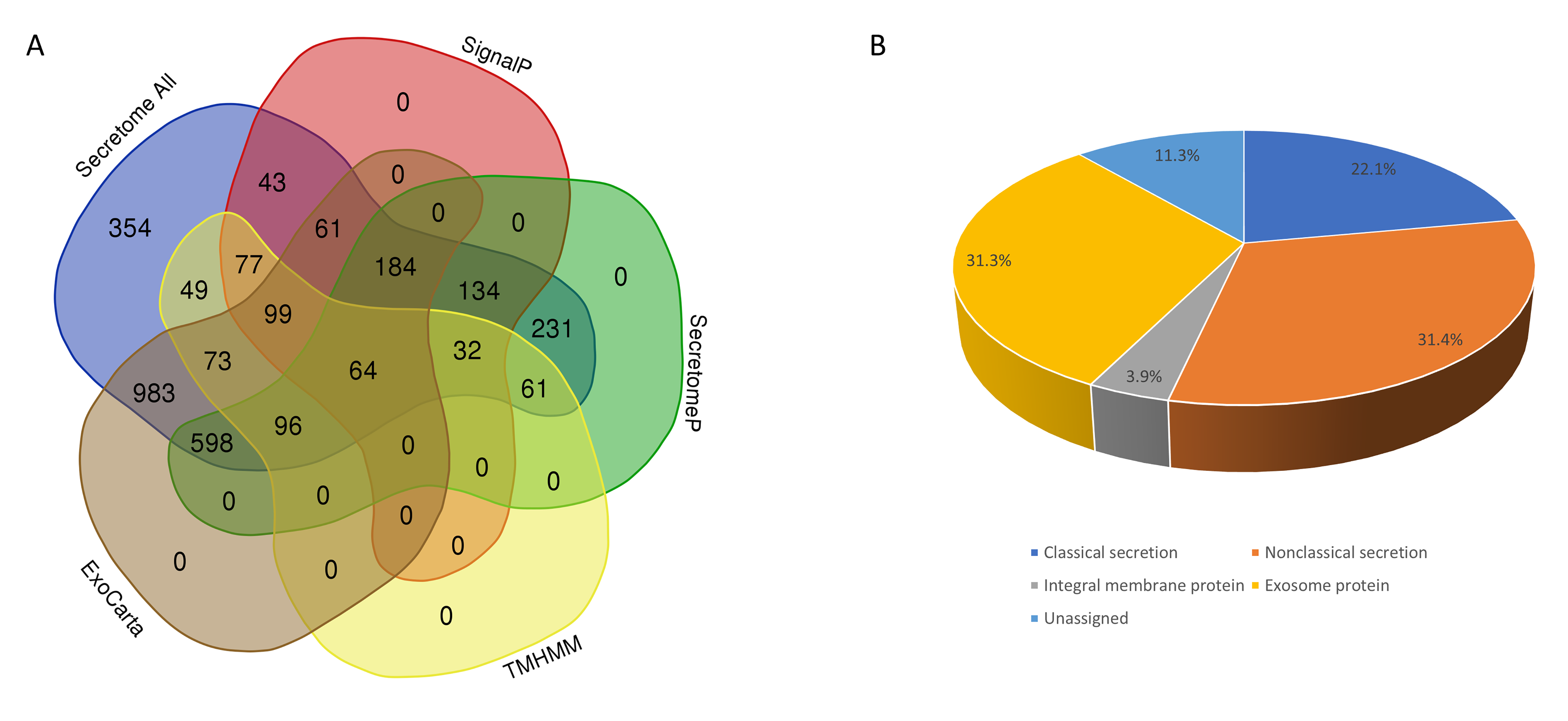


**Supplementary Figure 4**

Distribution of protein intensity (LFQ) from profiling experiments of cellular proteome (A) or secretome (B).


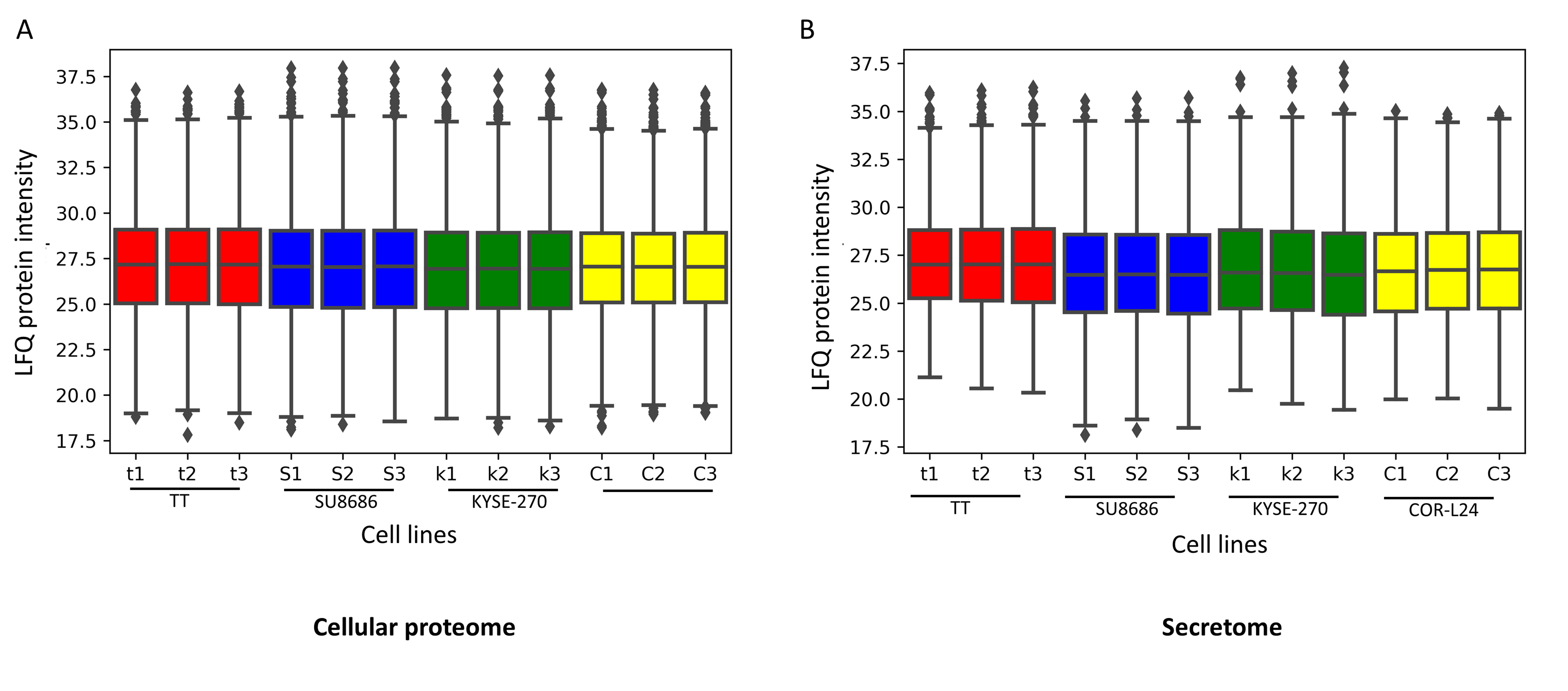


**Supplementary Figure 5**

Dynamic range of the composite cell line cellular proteome estimated by iBAQ.


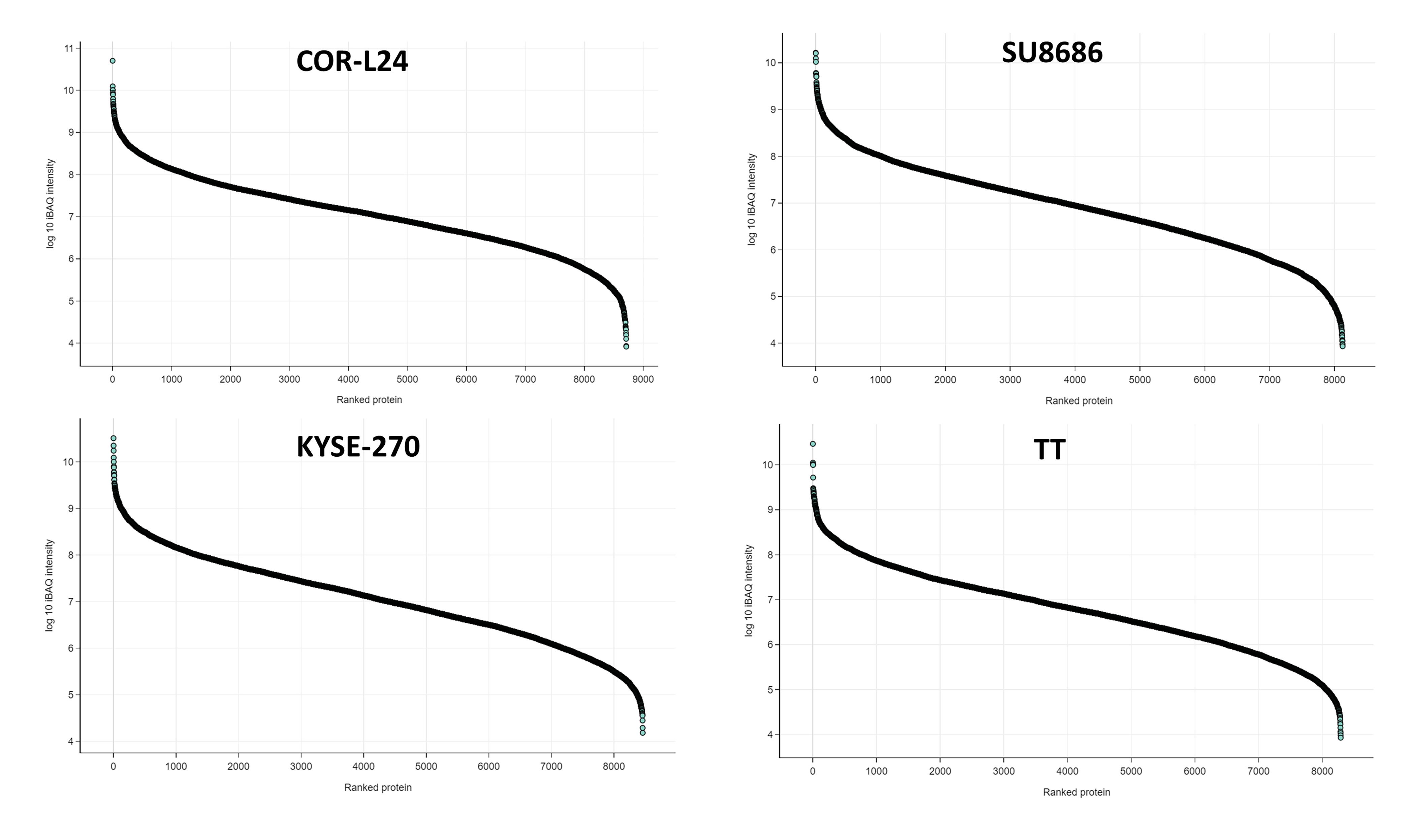


**Supplementary Figure 6**

Dynamic range of the composite cell line secretome estimated by iBAQ.


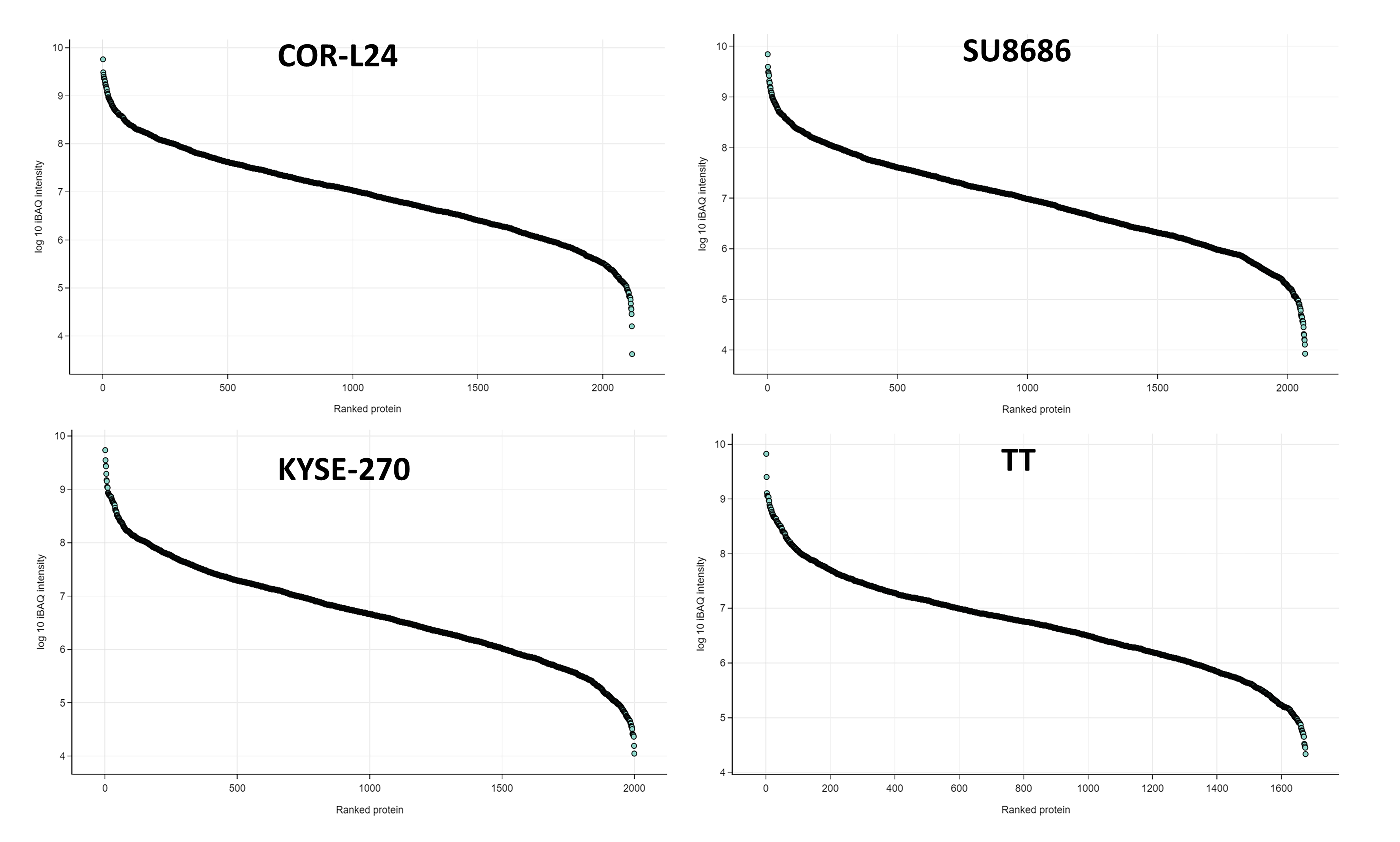


**Supplementary Figure 7**

Distribution of the median logarithmic protein intensity (iBAQ) of all proteins quantified in cellular proteome (A) or secretome (B).


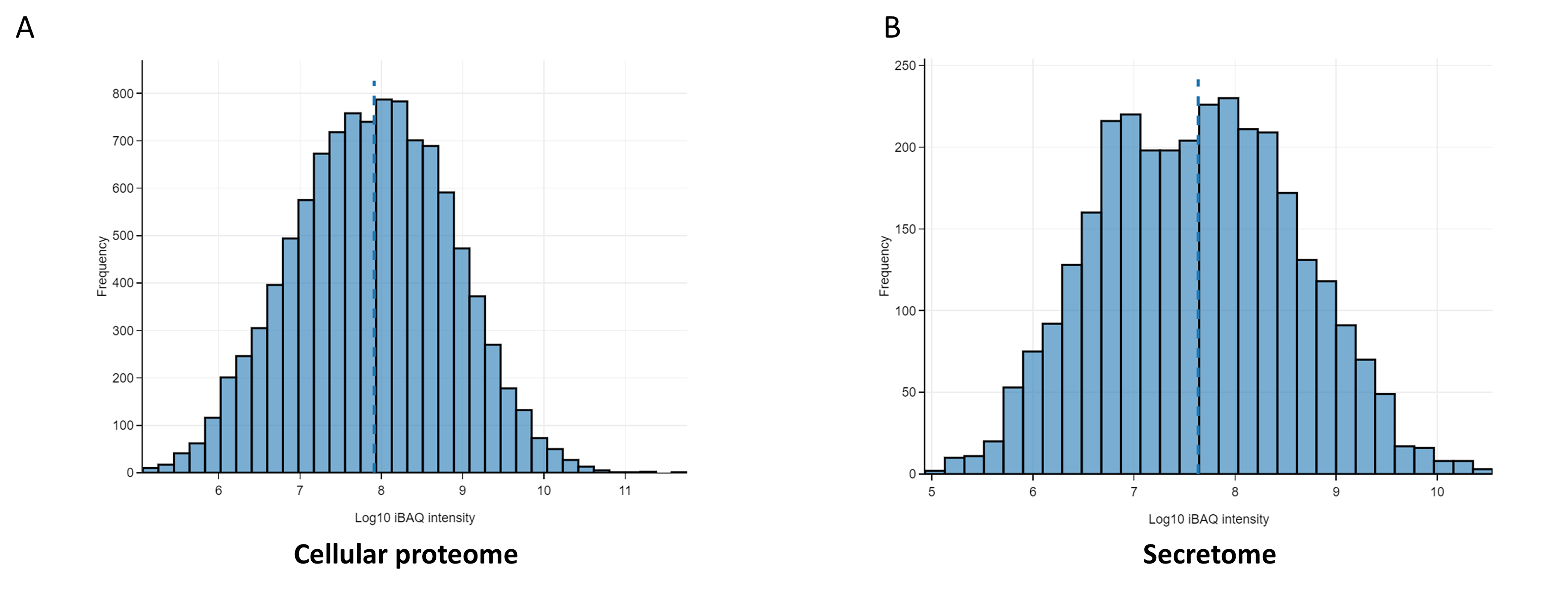


**Supplementary Figure 8**

Matrix representation of scatter plots and Pearson correlation values of the label-free protein abundances of triplicates of each cell line cellular proteome(A) and secretome (B) against the others. Missing values were imputed based on a normal distribution (width = 0.3, down-shift =1.8)


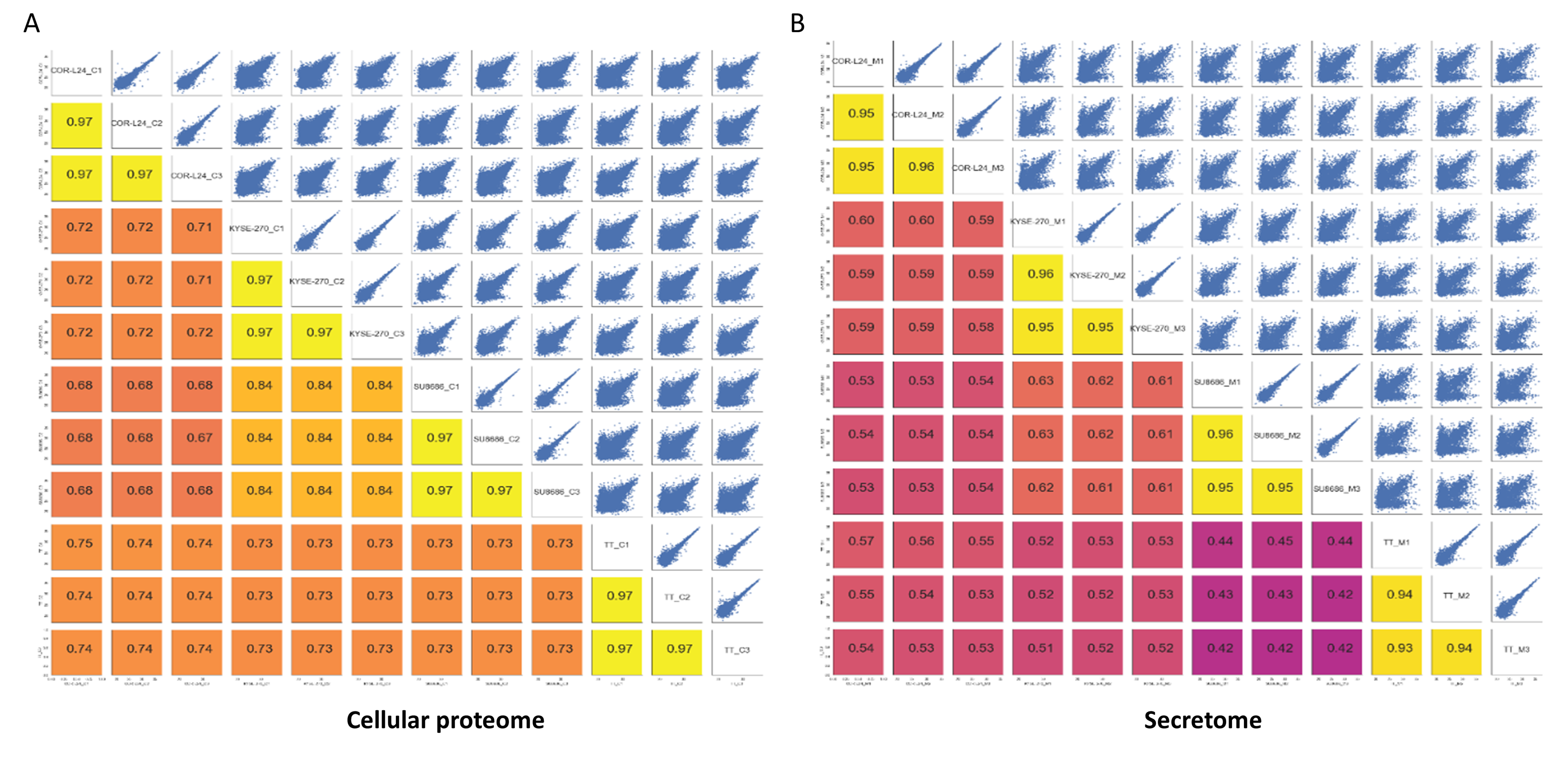


**Supplementary Figure 9**

PCA and unsupervised hierarchical clustering based on label-free proteome quantification in cellular proteome (A and C) and secretome (B and D).


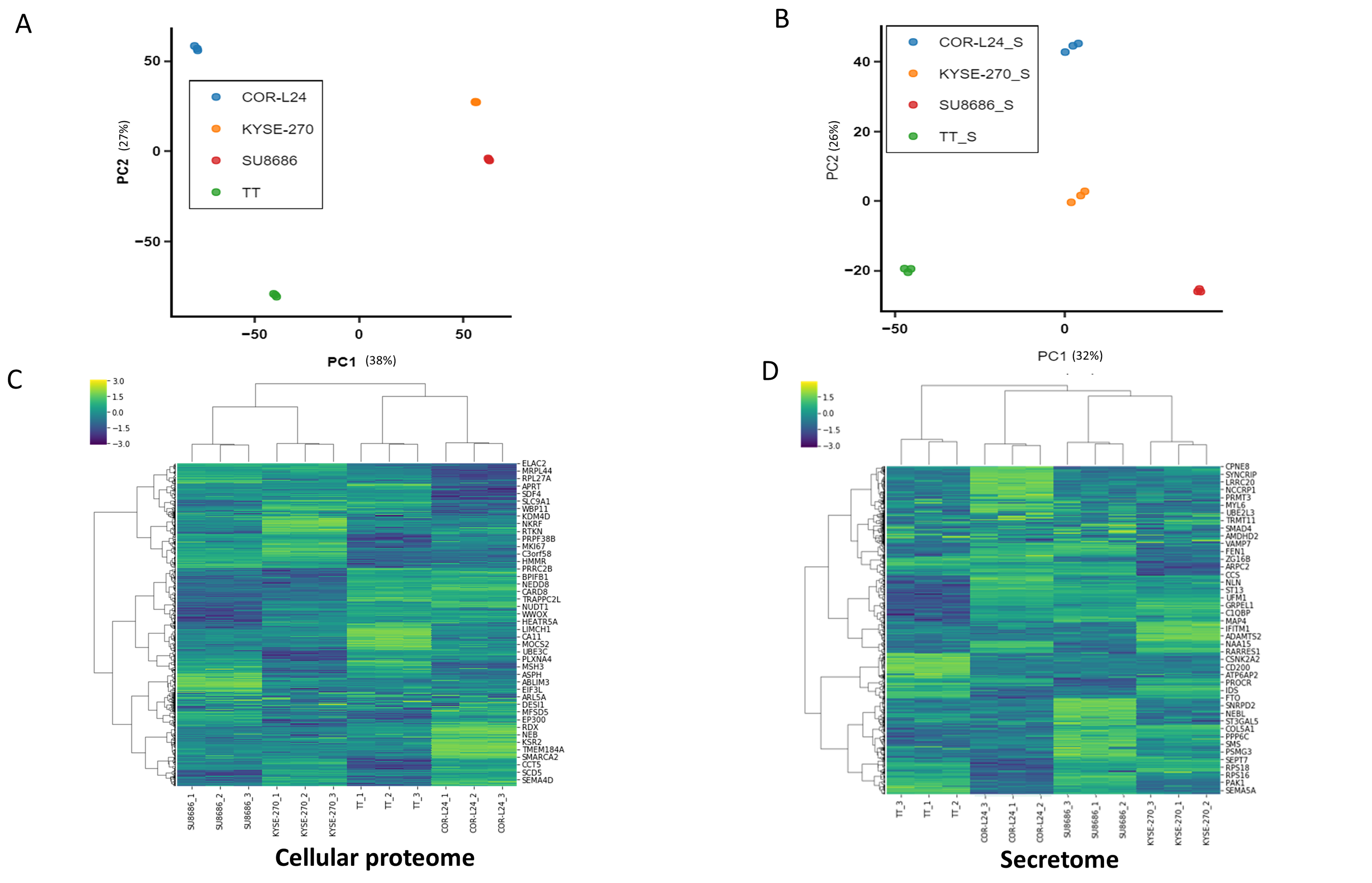


**Supplementary Figure 10**

Safety-related genes across organ classes.


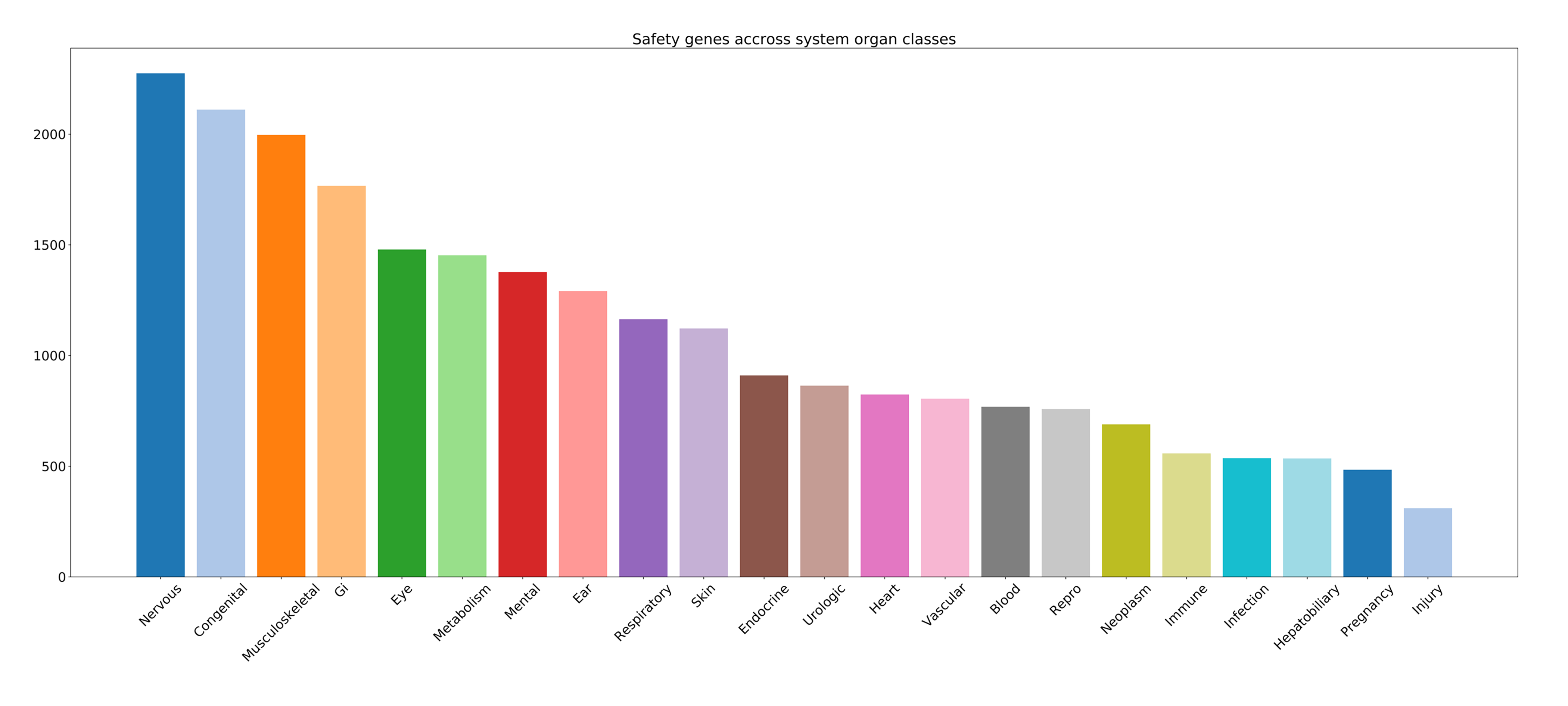

Supplement: Supplementary file 2 — Supplementary Information 2. [file 41598_2021_95354_MOESM2_ESM.docx]
